# Case Report: Abnormal ECG and Pantalgia in a Patient With Guillain–Barré Syndrome

**DOI:** 10.3389/fcvm.2021.742740

**Published:** 2021-10-06

**Authors:** Xiangqi Cao, Manyun Tang, Hui Liu, Xin Yue, Guogang Luo, Yang Yan

**Affiliations:** ^1^Stroke Centre and Department of Neurology, The First Affiliated Hospital of Xi'an Jiaotong University, Xi'an, China; ^2^Department of Cardiovascular Medicine, The First Affiliated Hospital of Xi'an Jiaotong University, Xi'an, China; ^3^The Biobank of The First Affiliated Hospital of Xi'an Jiaotong University, Xi'an, China; ^4^Department of Cardiology, Cedars-Sinai Medical Center, Smidt Heart Institute, Los Angeles, CA, United States; ^5^Department of Cardiovascular Surgery, The First Affiliated Hospital of Xi'an Jiaotong University, Xi'an, China

**Keywords:** Guillain-Barré syndrome, pantalgia, electrocardiogram, transitional T-wave inversion, acute coronary syndrome

## Abstract

**Background:** Guillain–Barré syndrome (GBS) is an acute immune-mediated disorder in the peripheral nervous system (PNS) characterized by symmetrical limb weakness, sensory disturbances, and clinically absent or decreased reflexes. Pantalgia and dysautonomia, including cardiovascular abnormalities, are common findings in the spectrum of GBS. It is usually challenging to distinguish GBS-related electrocardiogram (ECG) abnormities and chest pain from acute coronary syndrome (ACS) in patients with GBS due to the similar clinical symptom and ECG characteristics. Here, we present a case of GBS complicating ACS.

**Case Summary:** A 37-year-old woman with a 2-month history of GBS presented to the emergency department due to pantalgia. The ECG showed a pattern of transitional T-wave inversion in the leads I, aVL, and V2 through V4 and shortly returned to normal, which appeared several times in a short time, but lab testing was unremarkable. Then, a further coronary computed tomography angiography (CTA) revealed the presence of critical stenosis of the left anterior descending artery, leading to the diagnosis of ACS. During the follow-up, she suffered from a non-ST-elevation myocardial infarction and accepted revascularization of the left anterior descending artery in the second week after discharge.

**Conclusion:** Guillain–Barré syndrome could accompany chest pain and abnormalities on ECG. Meanwhile, it is essential to bear in mind that “GBS-related ECG abnormalities and chest pain” is a diagnosis of exclusion that can only be considered after excluding coronary artery disease, especially when concomitant chest pain, despite being a common presentation of pantalgia, occurs.

## Introduction

Guillain–Barré syndrome (GBS) is an immune-mediated disorder characterized by acute demyelinating polyradiculoneuropathy in the peripheral nervous system (PNS), which presents as symmetrical limb weakness with absent or reduced reflexes in the lower limbs and ascending gradually in a majority of patients (1–3). Neuropathic pain (NP) is also a common presentation in patients with GBS, which was reported in 66% patients in the acute phase (4), mainly including radicular pain, meningism, painful paresthesias, muscle pain, and arthralgias. In addition, a variety of abnormalities on electrocardiograms (ECG) including T-wave inversion can be observed on ECG due to autonomic dysfunction during the course of GBS (1, 5, 6). Thus, it is usually challenging for clinicians to distinguish GBS-related chest pain and ECG abnormities from acute coronary syndrome (ACS) in patients with GBS due to the similar clinical symptoms and ECG characteristics. Here, we present a case of GBS complicating ACS.

## Case Presentation

A 37-year-old woman presented to the emergency department due to pantalgia for 1 day. She reported symmetrical limb pain, chest pain, and back pain at rest, without radiation. There was no dyspnea, nausea, vomiting, or diaphoresis. She denied medical history such as hypertension and diabetes mellitus and special personal history.

The patient was admitted to an outside hospital with a chief complaint of limb weakness and paresthesia 2 months ago. Significant findings were not seen in the cerebrospinal fluid obtained by lumbar puncture, and her laboratory tests were normal. A diagnosis of GBS was made, supported by the evidence of symmetrical peripheral nerve injury involving the upper and lower extremities from electrophysiologic testing. Thereby, she received a standard intravenous immunoglobulin (IVIG) for five consecutive days.

**Figure 1:**
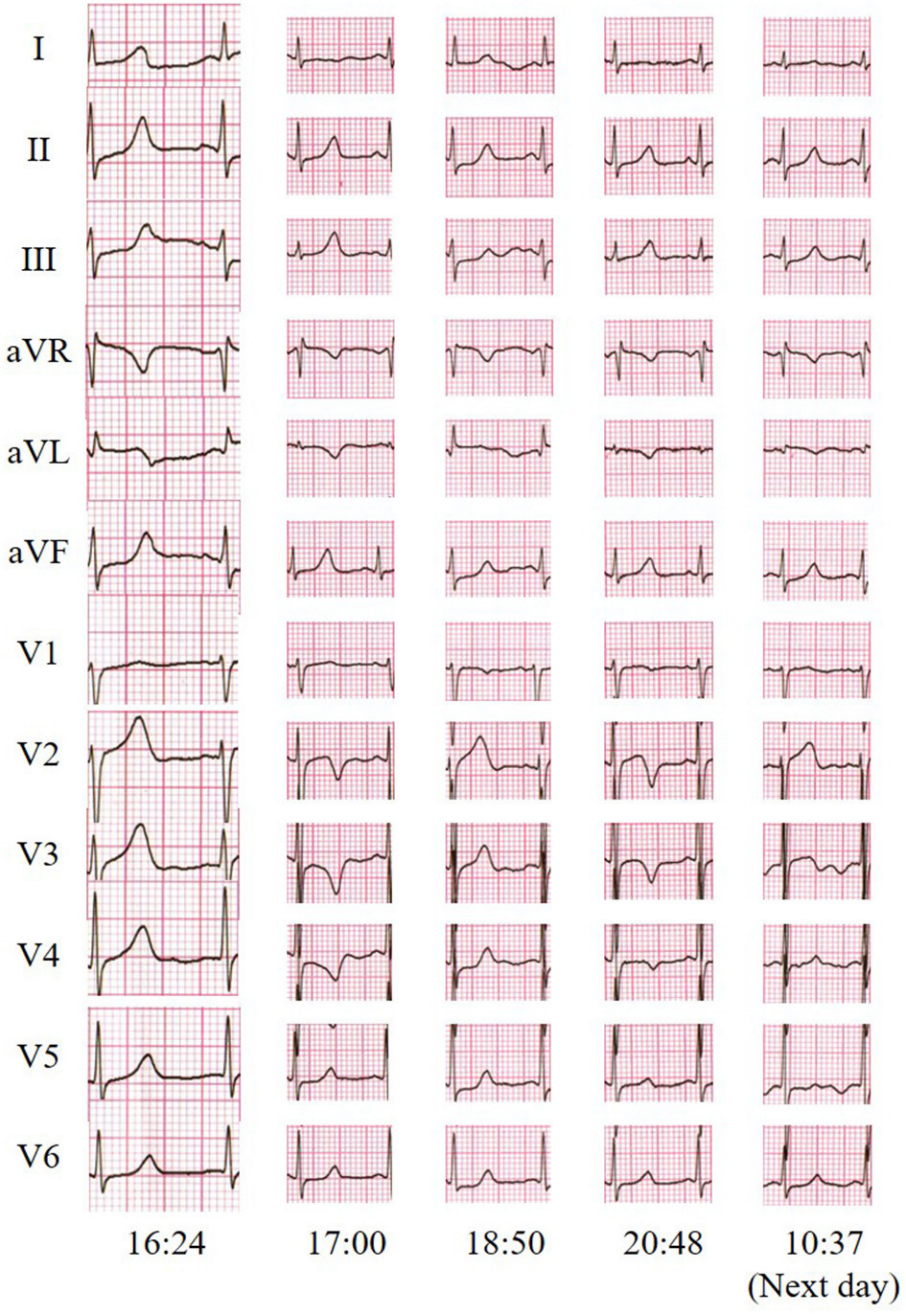
Twelve-lead electrocardiogram indicated transitional T-wave inversion in the leads I, aVL, and V2 through V4 and shortly returned to normal.

On arrival, the physical examination revealed blood pressure of 121/82 mmHg, heart rate of 88 bpm, respiratory rate of 20/min, and pulse oximetry of 100% on ambient air. Neurological examination mainly showed that the motor strength was 3/5 in her upper extremities and 2/5 in her lower extremities, accompanied with decreased reflexes, while other physical examinations were normal. The ECG taken at the emergency department showed a pattern of transitional T-wave inversion in the leads I, aVL, and V2 through V4 and shortly returned to normal. This ECG pattern appeared several times in a short time (Figure 1). Lab testing, including cardiac injury biomarkers, electrolytes, and echocardiography, was unremarkable.

**Figure 2:**
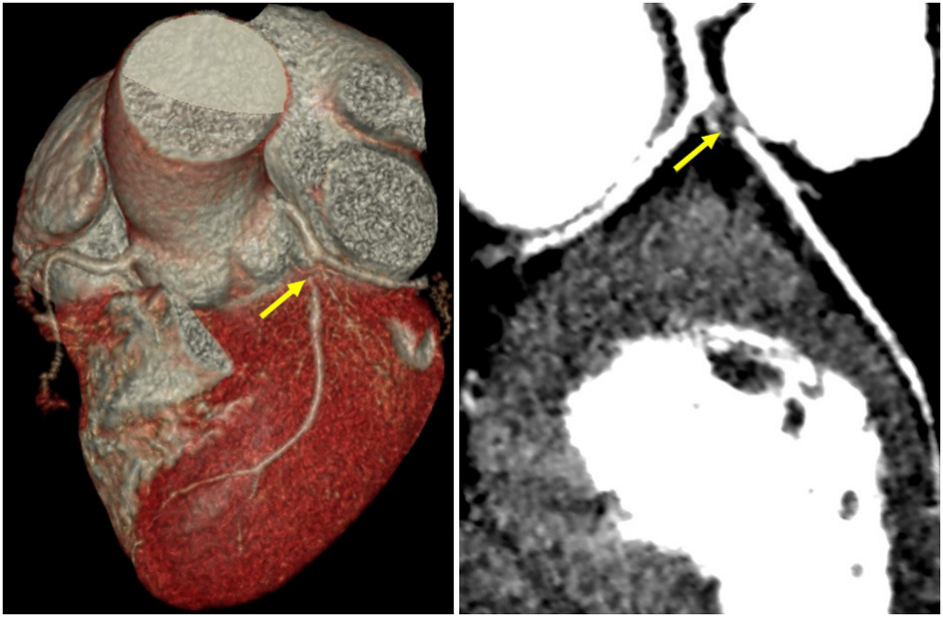
Coronary CT angiography revealed critical stenosis of the left anterior descending artery (yellow arrows).

Guillain–Barré syndrome-related ECG abnormities were initially suspected in such a young woman without any risk factors for heart disease, but ACS-induced ECG abnormities could not be completely ruled out. A further coronary computed tomography angiography (CTA) was performed and revealed critical stenosis of the left anterior descending artery (Figure 2), leading to the diagnosis of ACS. However, this patient who was menstruating refused coronary angiogram (CAG) and revascularization due to the risk concern. She was then discharged after receiving guided pharmacologic therapy.

Two weeks after discharge, unfortunately, she was transferred to the emergency department again for non-ST-elevation myocardial infarction. Emergent CAG and revascularization of the left anterior descending artery were performed. The patient did not complain of obvious dysautonomia such as abnormal blood pressure fluctuations, gastric dysmotility, urinary retention, and hyperhidrosis at the scheduled follow-up visit 3 months after discharge. Her follow-up continues under the care of a multidisciplinary team of cardiologists, neurologists, psychologists, and others.

## Discussion

Autonomic dysfunction involving cardiovascular abnormalities during the course of GBS is universally acknowledged (1, 5, 7–9), and it is suggested that T-wave inversion can be observed in 12.5% of patients with GBS (5). Furthermore, pantalgia, including atypical chest pain, is a common and often severe symptom in the whole spectrum of GBS (4, 9).

Abnormal ECG and chest pain manifestations are the most used diagnostic criteria of ACS. In clinical practices, however, acute GBS patients with abnormal ECG or chest pain still face a diagnostic dilemma. First, GBS-related ECG patterns often show varied abnormities, of which sinus tachycardia is the most common pattern ranging from 100 to 120 bpm that normally does not require any treatment (10), followed by ST-T changes (elevation, inversion, or depression). Other abnormities, such as sinus bradycardia, are also observed (1, 5, 6). These abnormal patterns could, in part, be explained by catecholamine-associated myocardial injury theories: a disorder of catecholamine uptake around myocytes (11), redistribution of coronary blood flow (12), or denervation hypersensitivity of the myocardium (6). As for the symptoms in the whole spectrum of GBS, pain is the most complex, and the underlying pathophysiology is largely unknown. Chest and back pain mimicking ACS are often considered to be caused by radicular nociceptive nerve pain—one of the GBS classic symptoms rather than the coronary arterial stenosis. In addition, the high prevalence of depression or anxiety in GBS patients can act as the predisposing factors of the pantalgia, including chest pain, which pose further challenges in diagnosis (13).

Acute coronary syndrome should also be considered in patients with GBS especially when presenting with concomitant chest pain and ECG abnormalities. This opinion can be partially supported by the fact that autonomic dysfunction during the GBS course often involves the heart, and cardiovascular diseases are important causes of morbidity and mortality in patients with GBS (2, 6). An observational study reported that ACS developed in 2.08% of patients with GBS (5), which probably could attribute to increased risk of prothrombotic state induced by immunoglobulin and corticosteroids therapy (14). Coronary vasospasm in GBS patients, as a result of dysautonomia accompanied by catecholamine rising, also contributes to ACS. In addition, Takotsubo cardiomyopathy (TC), a rare complication of acute GBS, could induce concomitant ECG abnormalities and chest pain (14). Takotsubo cardiomyopathy is a reversible cardiac dysfunction demonstrating a transient wall motion abnormality with or without apical involvement in the absence of obstructive coronary artery disease (15). The pathophysiology of TC remains unclear, but it seems to be from catecholamine release secondary to the sympathetic excitation of the brain, inducing hyperdynamic basal contraction and apical systolic dysfunction (15). All of these abnormalities are often reversible in days or weeks with GBS therapy but can be responsible for complications. Therefore, TC in this patient we described above here cannot be excluded even if the echocardiography was unremarkable. Moreover, coronary artery disease and GBS are considered to share the common risk factors and mechanisms, and coronary artery disease may predate acute GBS; however, this could not be confirmed or disproved until CTA or CAG. In this case we presented, the patient was finally diagnosed as ACS after CTA result showing critical stenosis of the left anterior descending artery. This case reminds us that “GBS-related ECG abnormities” is a diagnosis of exclusion that can only be considered after excluding coronary artery diseases, especially when concomitant chest pain (despite being a common presentation of pantalgia) occurs.

## Limitations

Several limitations of this study should be acknowledged. As a matter of fact, it could not be confirmed whether the ECG abnormities with chest pain were due to ACS or GBS in this patient. The ECG abnormities could be caused by any one of the two diseases, even two of them concomitantly. Based on the further results of CTA and CAG, ACS-induced ECG abnormities were strongly suspected and later supported by clinical follow-up, but GBS-related ECG abnormities could not be excluded completely. Besides, the patient accepted CAG and revascularization in another hospital, and we could not acquire the details of the CAG and revascularization results.

## Conclusion

Generally, the ECG abnormalities detected in GBS patients can be attributed to autonomic dysfunction. However, when there is concomitant chest pain and ECG abnormalities resembling the ECG pattern of myocardial ischemia, other conditions should be considered, and CTA is recommended to rule out coronary artery disease. It is essential to bear in mind that “GBS-related ECG abnormalities and chest pain” is a diagnosis of exclusion that can only be considered after excluding coronary artery diseases.

## Data Availability Statement

The raw data supporting the conclusions of this article will be made available by the authors, without undue reservation.

## Ethics Statement

Written informed consent was obtained from the individual(s) for the publication of any potentially identifiable images or data included in this article.

## Author Contributions

GL, YY, and XC contributed in this patient care, diagnosis, and treatment. XC and MT collected the data and drafted this manuscript. YY, GL, HL, and XY revised the final version of the manuscript. All authors have read and agreed to the published version of the manuscript.

## Conflict of Interest

The authors declare that the research was conducted in the absence of any commercial or financial relationships that could be construed as a potential conflict of interest.

## Publisher's Note

All claims expressed in this article are solely those of the authors and do not necessarily represent those of their affiliated organizations, or those of the publisher, the editors and the reviewers. Any product that may be evaluated in this article, or claim that may be made by its manufacturer, is not guaranteed or endorsed by the publisher.
